# Supplementary material for: Patterns of Intron Gain and Loss in Fungi
Source: PLoS Biol. 2004 Nov 30;2(12):e422. doi: 10.1371/journal.pbio.0020422 (PMC532390; doi:10.1371/journal.pbio.0020422)
Supplement: Table S1 — Also available at http://genes.mit.edu/NielsenEtAl/. (4.3 MB ZIP). [file pbio.0020422.st001.zip › NielsenEtAl/html/166.html]

AN1018.1.NCU07023.1.MG01823.1.FG09615.1


```
 CLUSTAL W (1.82) Multiple Sequence Alignments - Introns Inserted


Sequence 1: NCU07023.1	908 aa
Sequence 2: FG09615.1	735 aa
Sequence 3: MG01823.1	804 aa
Sequence 4: AN1018.1	801 aa
Alignment Length: 970 aa
Number Identitical Residues: 264 aa
Alignment Score (without introns) 13721


MG01823.1 	MAVSTGGAEDLMGNGNGTES-------------SRRSSDSMMRSDFVPLVAVVDFHHAR~
NCU07023.1	MS-------------------------------VPPTPTTERKPGFIPLVAVVDFHHAR~
FG09615.1 	MSSDESPPVESPIGLGAPPPESNDDLKSILSPILPSSPAPFNRADFIPLVTVVGFHHAR~
AN1018.1  	--------------------------------------MSHTRVYKDPIVLVIDFHHAR2
          	                                       .  :    *:* *:.***** 

MG01823.1 	GPEVERWFGAPEGFDPAVEYDWSLLPFMALSDGAHA2GCRRSNCSSTEEFSYFTLLRPAT
NCU07023.1	GPEVERWFGVPEDSDPAAEYDWGLLPFMALSDGAHA2--------ATEDFSYFTLLRPAT
FG09615.1 	GPEVENWFGAQEGTDPAADYGWSLLPFMALSDGAHA2--------SEEDFSYFTLLRPET
AN1018.1  	GPEIELCIG-PEGTDPAAENDWSLLPFMALSDGAHM2--------STEEFSYFTLRRRGT
          	***:*  :*  *. ***.: .*.************          : *:****** *  *

MG01823.1 	EASP-ATSLFGIACTRQMDSSQLINRPADVTRSTVQKAVVVIADHPRSFGMLRERMSIIT
NCU07023.1	ASEPEATSLFGISCTRQMDASQLLNRPADVTRSTVQKAVVIIADSPQYFGMLRERLSVVT
FG09615.1 	ETKP-ATSLFGISCTRQLDSTQLINRPADVTRSTVQKAVVVIADSPQFFGMLRERLSIVT
AN1018.1  	DTEP-PTSLFGIACSRQIDSNALIYKPPDVTRSTVQKAVVVVTENPKCLGQLREKLSVVT
          	 :.* .******:*:**:*:. *: :*.************:::: *: :* ***::*::*

MG01823.1 	KAWFDQR~EFTDVDILR~RFQESLAEDKGRGRLAS-EDEDKDTYLG~LSLRELVREFKWQ
NCU07023.1	KAWFAQR~EFTDVEILR~RFQESLADEKARGTLLGEKEEDRDLYLG~MSLRELVREFRWQ
FG09615.1 	QAWFAQR~EFTDVEILR~RFQESLADEKMKGVLN--DQVDRDQYLG~MSLRELIHEYKWQ
AN1018.1  	SAWFAQR2DFSDLDILK0KFREGLVISLQK------DEGFKDQTLG1LSLREMIHDYKYQ
          	.*** ** :*:*::**: :*:*.*. .  :      .:  :*  ** :****:::::::*

MG01823.1 	TLVLFKCCLLQP-~--------------K0FSLISLIPGLLRHLQDSASPELHSYEDRLS
NCU07023.1	TLVLLKCCLLQPK~MLFFGSRCERLCMMQ~FSLISLIPGLLRNLQDSAGPELDNYEKKLQ
FG09615.1 	TLVLLKCCLLQPK0MLFFGSRCDKLCMMQ~FSLLSLIPGLIRNLQDSADPELNSYETSLS
AN1018.1  	TLVLFKALLLQPK0MLFFGSRCERLCMIQ~FSLVSLIPGLMNHLQDCADPSFDTYAQTVK
          	****:*. ****.     .: ..  .  : ***:******:.:***.*.*.:..*   :.

MG01823.1 	KPTGVKTSDRTSL~LAYMGMPLQIFGK0------GSLFGPYTPLQQLDILADHGTKSYIV
NCU07023.1	RPTSLRTSDRNSL~LAYMGLPLQIFGK~------GSLFGPYTPLQQLDILADFGTKSYIV
FG09615.1 	KPTSLRTSDRNSL~LCYMGLPLQIFGK0QLTWKQGSLFGPYTPLQQLDILADFGTKSYIV
AN1018.1  	KPTSLKTSDRSSL1LAYMGLPLQIFGK0------GSMFGPYTPLQQLDLLADDGTKSYVV
          	:**.::****.** *.***:*******       **:***********:*** *****:*

MG01823.1 	GSTNSILLQQKDRYSDILID~LDHNSVSITSQSLKSALSLSSADRRWIESVAAEVNDTWD
NCU07023.1	GSTNSLLLQQKDRYSDILIN~LDEDSINITSPSLKSALALSTPDRRWIDFITQNVNDTWD
FG09615.1 	GSTNSLLLQQKDRYSDILIN~LDEESVVINSQQLRTALTLTAADRRWIDYLTHEINETWD
AN1018.1  	GSTNSLLLQQKDRYSDILIN0LDEDTINITSSSLRNALTLSVADRRWIDAITQIVNDTWD
          	*****:*************: **.::: *.* .*:.**:*: .*****: ::  :*:***

MG01823.1 	EANPGRPKTMGYRGSEESIRLQFEDYMLSLMAAVKCRNWQAKHKDNP-----RMQLS--H
NCU07023.1	DANPGMPKTMGYVGSEEFIRLQFEEYLLSLISSVKYHNHLAIHSQNP-----RMLLP--H
FG09615.1 	DANPNRPKTLKYVGSEEFIRLQFEEYILALISSVKYHNYLTSNPNNP-----KAILP--E
AN1018.1  	EAHPNQPKTHGYMGSEEFIRLQFEEYLLALLSCMKYHEELNSYLTGEPSRRNRAQLDSYN
          	:*:*. ***  * **** ******:*:*:*::.:* ::       . .:  .:  * : .

MG01823.1 	VEGDPSVDFNPDWIEHWMRTDNYRMWNTYT-DDCLFDIAEPKHPCASGLS------RLAQ
NCU07023.1	IEGDPSLDFNADFIEAWKRTENYRIWDGHT-DSHLFDIVEPKHPCAGGLTIDDVQRRIAQ
FG09615.1 	VEGDPVADFGYEWVEAWKRTENYRMWNSHT-DSHLFDIVDPRHPCAGGLNIEDVQRRIAE
AN1018.1  	IEGDPALEFNQEFLAHWRTTSNYALFQRLTSDALLFSVAEPRHPSAGGLTIDDVQRRISQ
          	:****  :*. :::  *  *.** :::  *:*  **.:.:*:**.*.**. .. . *:::

MG01823.1 	QVQDLGLDERFAAAGVVVGRGVAAGKERASTILNKLYSDVEALREAQRKKAEQQQQQAKD
NCU07023.1	QVQDLHLDERFAVGKEVLGRNLAAGKEKASTMFNKLYADMEALREQRRKAAEEAEKQRQA
FG09615.1 	QVKELHLDERFAQGREVLGRNLAAGREKASFVLNKLYADMEALREAQRRRAEEAR----A
AN1018.1  	QVADLHLDEKVREGREALNRHLSTGQKKVSAAFNSFWADIEAMREAQRKRNEEKAATQSP
          	** :* ***:.  .  .:.* :::*:::.*  :*.:::*:**:** :*:  *:     . 

MG01823.1 	NGNGTARNSQDLSGVTRAADLSKS--------------------AQPAQTVGARAGAYAS
NCU07023.1	EAAAVGNGQQHGSEKGGSSGSGNGNGGNGQGG---------LAPAAAVSTVGSKAGAFVS
FG09615.1 	ASPPDASGQSTGSDASRAGQTG--------------------------QSAGARAGAYIG
AN1018.1  	RPSLDRSSTLPTSHSDSTSNSSTSASSWFPNRKVAVPAVDMTQAQASVSAVGQKAGAYFS
          	       .    *    :.  ... ..   . . : .: .   .  . .:.* :***: .

MG01823.1 	SWASWAGEKRKAWAR--------------------------SSSNTAASP----------
NCU07023.1	SWTAWAGEKRKGWGRSAPSTPITETAPVPPTTTTDTTTTSDTSSQTGSQQPEEKDKEKEQ
FG09615.1 	SWAAWAGEKRR------------------------------TSGWGG-------------
AN1018.1  	SWGTWASEKRKEWQE----------------------KKSASSSPTASAAVT--------
          	** :**.***:                          ..: :*.  .:            

MG01823.1 	---PAATTPTTATGGWSFVGRRSSKRDTTSSLASSR------------------LSYSDV
NCU07023.1	EKKGWGWSKAIRNRTSLLLSGGSTSEERETFMPQSPPRNGAYAALPLGTGSPESVRTTRT
FG09615.1 	---GWGRKANTKPDKSADNSTSSSPIDRDYQMISAP--------------------MSRG
AN1018.1  	---SPSTPTLASTTESIDTTNTNTDKDRRQSFQSFR--------------SEASSTISRS
          	     .                .:  :    : .                :  :   :  

MG01823.1 	NSETEFNLRSPTASVASSGVHVDAQPTPPSD-------------------------VSIP
NCU07023.1	GNSSTVEQRAYRRRALSGESMLDAVEGEDGEGFSGSEFGSPERVRGGIARKPVGGVGSRP
FG09615.1 	GSSDEPGPRTQRGASFS-ESILSGVSEPN----------------------------SRP
AN1018.1  	GSRRK-RLSNLFFKRESLDFNVGAISRDGDD-----------------------HETVYP
          	..              *    :..     ..                            *

MG01823.1 	TSPAAEKR-----WMFSPEVGGSSSGNLKG-----LNDGATLVPSTAVTPTTDAANEVLF
NCU07023.1	GTAGTAAGSVNLMSLASPEVVRSANNTKTTITTQEENAGETGVKKDEKVEIEDKSDKADK
FG09615.1 	-TSGTNK------PLPGPPVPKKE---------------DDGFQTEGVVGSKDKLAGAST
AN1018.1  	KSPLSREE-----SILGDDIPETSAEQYKG-------------ERKLDIHPKTEADVAER
          	 :. :         : .  :  .     .                            .  

MG01823.1 	QAPAASSVSISAAQAGPDKPDEEEEEGEES----KPNLFANIETENVWASDTPSSPILLR
NCU07023.1	EDKGMQEVSLDDTPATATEHVKMEPSPVLAPITTTTTTTTTEETQKEGSRSRSPSPSPLQ
FG09615.1 	HPDGNGAAAKD-------------------------------------------------
AN1018.1  	KDTTVNNVPSDGTRTPPPQQ--------------PAAALDSIELEPRANLPGSESEKVVV
          	.      .. . : : . .                .    . . .       . :     

MG01823.1 	KPSPEPKASAPVGSKIVFLP
NCU07023.1	SPAVIVAAEEAAKAKEVWDK
FG09615.1 	--------------------
AN1018.1  	QSSKSTNENEPPLK------
          	..:     . .
```
